# Managers, modelers, and measuring the impact of species distribution model uncertainty on marine zoning decisions

**DOI:** 10.1371/journal.pone.0204569

**Published:** 2018-10-10

**Authors:** Bryan Costa, Matthew Kendall, Steven McKagan

**Affiliations:** 1 NOAA National Centers for Coastal Ocean Science, Biogeography Branch, Silver Spring, Maryland, United States of America; 2 NOAA National Marine Fisheries Service, Habitat Conservation Division, Saipan, CNMI; Swansea University, UNITED KINGDOM

## Abstract

Marine managers routinely use spatial data to make decisions about their marine environment. Uncertainty associated with this spatial data can have profound impacts on these management decisions and their projected outcomes. Recent advances in modeling techniques, including species distribution models (SDMs), make it easier to generate continuous maps showing the uncertainty associated with spatial predictions and maps. However, SDM predictions and maps can be complex and nuanced. This complexity makes their use challenging for non-technical managers, preventing them from having the best available information to make decisions. To help bridge these communication and information gaps, we developed maps to illustrate how SDMs and associated uncertainty can be translated into readily usable products for managers. We also explicitly described the potential impacts of uncertainty on marine zoning decisions. This approach was applied to a case study in Saipan Lagoon, Commonwealth of the Northern Mariana Islands (CNMI). Managers in Saipan are interested in minimizing the potential impacts of personal watercraft (e.g., jet skis) on staghorn *Acropora* (i.e., *Acropora aspera*, *A*. *formosa*, and *A*. *pulchra*), which is an important coral assemblage in the lagoon. We used a recently completed SDM for staghorn *Acropora* to develop maps showing the sensitivity of zoning options to three different prediction and three different uncertainty thresholds (nine combinations total). Our analysis showed that the amount of area and geographic location of predicted staghorn *Acropora* presence changed based on these nine combinations. These dramatically different spatial patterns would have significant zoning implications when considering where to exclude and/or allow jet skis operations inside the lagoon. They also show that different uncertainty thresholds may lead managers to markedly different conclusions and courses of action. Defining acceptable levels of uncertainty upfront is critical for ensuring that managers can make more informed decisions, meet their marine resource goals and generate favorable outcomes for their stakeholders.

## Introduction

In environmental management, most decisions have uncertainty associated with them [1]. Uncertainty originates from two main sources, including deficiencies in the input data and partial confidence in the projected outcomes. Often, uncertainty from these different sources is not well defined or quantified. This forces managers to accept an implicit level of risk in their decision-making processes. There is an increasing body of evidence that suggests incorporating uncertainty may have profound effects in marine management decisions and on conservation outcomes [1, 2, 3, 4]. Understanding and including measures of uncertainty can help managers more confidently identify priority sites, adequately protect habitats, convey the range of potential outcomes, and ensure that limited resources are used as efficiently as possible [5, 6]. Ignoring uncertainty in management decisions may cause suboptimal outcomes that increase opportunity costs to the local community, unintentionally waste limited resources, or fail to meet conservation goals altogether [1].

For example in Fiji, the Government set a conservation goal to protect 30% of their key marine habitats by 2020, including intertidal areas, mangroves, seagrasses, soft-bottomed lagoons and coral reefs [7, 8]. Tulloch et al. 2013 [4] found that for a sub-region in Fiji, the potential location of these priority habitat conservation areas changed when uncertainty was considered in the decision making process. These locations changed because some key habitats were less accurately mapped than others (e.g., coral reef, accuracy = 0.52 vs. seagrass, accuracy = 0.99). This lower thematic accuracy meant that there was less certainty about where coral reefs were located, requiring the protection of larger amounts of mapped coral reef area to confidently meet the Government’s 30% conservation goal. This trend was similar across other high priority habitats (e.g., soft-bottomed lagoons, accuracy = 0.79). When trying to design an optimal reserve network, the uncertainty associated with these various habitats combined additively, increasing the overall size of the potential reserve network by 50% compared to other network configurations. The trade-off was that a larger reserve network allowed the Fijian government to be more certain (i.e., with a 90% confidence) that their 30% conservation targets were met for key habitats across the marine region.

While the results from Tulloch et al. 2013 [4] are telling, one key limitation of their analyses was their use of classified habitat maps (CHMs). Marine managers have long relied on CHMs to make their decisions because they distill complex spatial patterns in the marine environment into simple maps showing the spatial distribution (i.e., presence and/or abundance) of marine organisms. While CHMs are extremely useful, they also have their limitations for informing management decisions. Their discrete boundaries and homogenous polygons miss potentially important information about habitat gradients and transitions on the seafloor. Their habitat accuracies (when known) do not account for varying levels of uncertainty within each habitat class or within each polygon [4, 9]. Combined, these limitations prevent managers from fully understanding the uncertainty associated with the location of benthic habitats, and the impact of this uncertainty on their management decisions.

Recent advances in modeling techniques, including species distribution models (SDMs), now make it easier to address these limitations, and generate continuous maps of marine organism distributions and associated uncertainty. Broadly defined, SDMs predict the potential presence, abundance, or biomass of individual marine species or assemblages (including benthic habitats) in an area using environmental variables. These continuous predictive maps are customizable by changing prediction thresholds, and can be tailored to meet specific management needs or conservation goals. In some cases, it may be best to err on the side of caution, such as when protecting endangered organisms or economically important fish habitats. In other cases, it may be better to focus on core locations for monitoring or protection, when budgets are limited or enforcement is challenging. The ability to tailor these maps makes SDM outputs more flexible (than CHMs) for many marine management applications.

SDM outputs are also more flexible (than CHMs) because they can quantify uncertainty across geographic space. Here, “uncertainty” is defined as both the performance and precision of SDMs. Precision describes the variation in the prediction and the mathematical models on which it is based. Predictions that vary by larger amounts contain more uncertainty. Performance (i.e., accuracy) describes the number and magnitude of errors in the species or assemblage prediction relative to their observed distributions. Predictions that contain more errors and larger errors contain more uncertainty. Uncertainty occurs and may vary for several reasons in SDMs. These reasons include differences in mapping approaches, modeling frameworks, sample sizes, sampling biases, detection rates, missing information, and/or errors in the environmental predictor data [10, 11, 12]. These factors can combine to introduce uncertainties that interact and propagate through the modeling process, making it essential to understand and quantify uncertainty when using SDMs to make management decisions [10].

In the marine environment, a number of techniques have been used to incorporate uncertainty in management decisions [13, 14, 15, 16]. While these techniques may be useful, their methodologies are often complex, opaque and difficult for managers and other non-users to understand fully. Making information on which management decisions are based transparent and easily understood is critical for developing stakeholder support, buy-in, and ultimately acceptance [1, 4, 17, 18]. Outputs from SDMs have the same issues. Non-technical managers and non-experts often have difficulty using SDM outputs because there are many different modeling approaches, many different methods for customizing their outputs, and many other nuances that make their application challenging [18]. Given that marine managers and decision makers seldom have expertise in SDM, there is a growing need to more explicitly link model outputs with their intended management application [1, 18, 19, 20]. Scientists and modelers must try to translate SDMs and uncertainty estimates into products that can be easily understood and immediately useful for making real-world decisions about marine resources, zoning, and conservation [1,17].

Here, our objective is to help fill this need by: 1) illustrating how SDMs and associated uncertainty can be translated into products for a targeted management application, and 2) explicitly describing the potential impacts of uncertainty on a specific marine zoning decision. Our case study focused on minimizing the impacts of jet skis on staghorn *Acropora* corals inside Saipan Lagoon, Commonwealth of the Northern Mariana Islands (CNMI). In the CNMI, staghorn *Acropora* is a multi-species coral assemblage that includes *Acropora aspera*, *A*. *formosa*, and *A*. *pulchra*. This assemblage is of particular concern because it grows in shallow (<4 m) water, is sensitive to warming ocean temperatures, and has long-thin branches that are easily broken. Jet skis also operate in shallow waters, where they may directly and indirectly damage these fragile corals. Our goal was not to make a single zoning recommendation, but rather to use a recently completed SDM for staghorn *Acropora* [21] to show a suite of zoning options, and describe their sensitivity to three different thresholds for probability of occurrence and three thresholds for precision (nine combinations total). Local managers can use this sensitivity analysis along with other considerations (e.g., distance from hotels, safety of the jet ski operators and other users, the cost of enforcement, stakeholder support) to identify the best locations for these competing goals and activities.

## Methods

### Description of study area & management issue

The lagoon along the western shore of Saipan (bounding coordinates: 145° 41’ 2” E x 145° 48’ 2” E x 15° 16’ 44” N x 15° 7’ 3” N) encompasses a diverse coral reef ecosystem, including extensive seagrass beds and staghorn *Acropora* thickets. This diverse ecosystem helps attract around a half million tourists to the Island annually [22, 23] directly contributing approximately $424 million per year to the CNMI economy [23, 24]. The lagoon offers tourists abundant recreational opportunities, including snorkeling, diving, parasailing, kayaking, and jet skiing, as well as potentially using new technologies, like seabreachers and hydroflight devices [25]. The Saipan Lagoon Use Management Plan (SLUMP) recommends how to spatially zone and restrict these activities for the safety of operators and protection of the lagoons’ resources, including benthic habitats. The impact of jet skis on surrounding benthic habitats has not been well studied in the lagoon, and the impacts for most seabreacher and hydroflight devices are not well established beyond manufacturer safety standards. However, research from other parts of the world have indicated that motorized vehicles, including jet skis, can have both direct and indirect environmental impacts on marine animals and benthic communities [26, 27, 28].

Since jet skis often operate at fast speeds in shallow waters, their potential, direct impacts include groundings, increases in suspended sediments, and other physical damage to benthic organisms from their water jets or wakes [29, 30, 31]. Direct impacts from water jets or wakes may be more noticeable in a place like Saipan Lagoon, which has shallow depths and typically calm and clear waters because of its extensive barrier reef. In addition to direct impacts, jet skis may also have indirect impacts to the marine environment. The indirect impacts from jet skis include above and below water noise, which have elicited complaints from other recreational users [30] and disturbed marine and nearshore animals [26, 28, 32]. Two-stroke jet skis are also known to impair water quality by releasing their gasoline/oil fuel mixture into the water and atmosphere [26]. These chemicals can accumulate in marine animals that inhabit shallow areas with little tidal flushing, such as Saipan Lagoon [33], and in larger marine organisms higher up the food chain [26]. They can also reduce the survival of coral larvae, including *Acropora* species, leading to declines in coral recruitment [34, 35].

**Fig 1:**
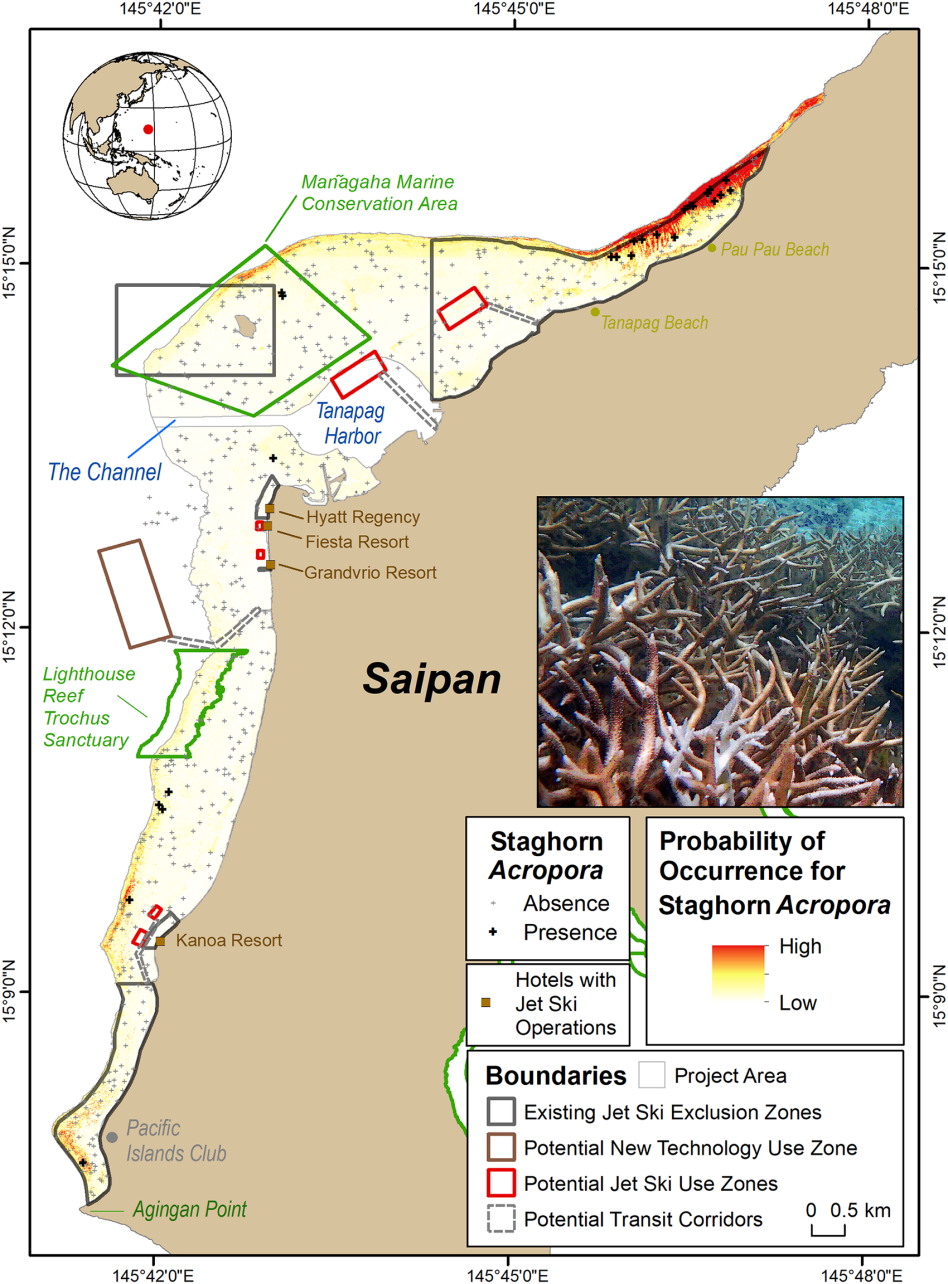
Key administrative boundaries in Saipan Lagoon. These boundaries include existing jet ski exclusion zones and potential jet ski operation zones, potential new technology (seabreachers, hydroflight devices) use zones, and transit corridors. These boundaries are overlaid on locations where staghorn *Acropora* (pictured above) has been documented, and where it is more likely to be found elsewhere in the Lagoon. The existing jet ski exclusion zones extend up to the reef crest, and appear offset because of the coarse scale at which they were digitized.

Staghorn *Acropora* corals are foundational species that form extensive coral thickets in the lagoon (Fig 1), supporting many commercially and ecologically important fishes and invertebrates. These corals, some of which (*A*. *aspera*) were considered for listing under the US Endangered Species Act [36], have recently experienced catastrophic losses throughout the region due to warming ocean temperatures [37, 38]. The ecological importance of staghorn *Acropora* and its decline has made zoning in the lagoon, particularly for the use of jet skis, an important local issue for managers in the CNMI [39]. Currently, there are six management zones in the lagoon where the operation of jet skis is prohibited by both commercial and recreational users. These six zones were based on past benthic habitat maps [40] and stakeholder considerations. New SDMs and maps showing the distribution of staghorn *Acropora* were developed recently for the lagoon [21]. These maps are more spatially resolved than those developed in 2008 [40]. With these new and improved maps, local managers may consider changing existing jet ski exclusion zones to better protect staghorn *Acropora* and improve the safety of jet ski operators.

In Saipan (like in other parts of the world), the goal of marine managers is to balance multiple community interests including: maximizing economic benefits, honoring existing practices, ensuring public safety, promoting sustainable practices, and minimizing potential impacts to the marine environment [41]. Prohibiting jet ski use in too large of an area may unnecessarily and negatively impact the commercial jet ski industry, which brings in an estimated $1.5 to $2.7M annually [39]. In 2006, over 12% (60,000) of Saipan’s tourists reported jet skiing while on the island. Conversely, allowing unrestricted access may create safety risks and negatively and irreversibly impact an already imperiled coral community. To better understand this and other issues in the lagoon, the CNMI Government is conducting a new economic valuation study [42] and has recently updated its SLUMP [43, 44]. Updated maps showing the spatial distribution of important benthic habitats, and the potential human activities taking place within them, are needed by managers to evaluate zoning scenarios that balance these multiple considerations [39, 43, 45].

### Predicting staghorn *Acropora* distributions in the Lagoon

**Fig 2:**
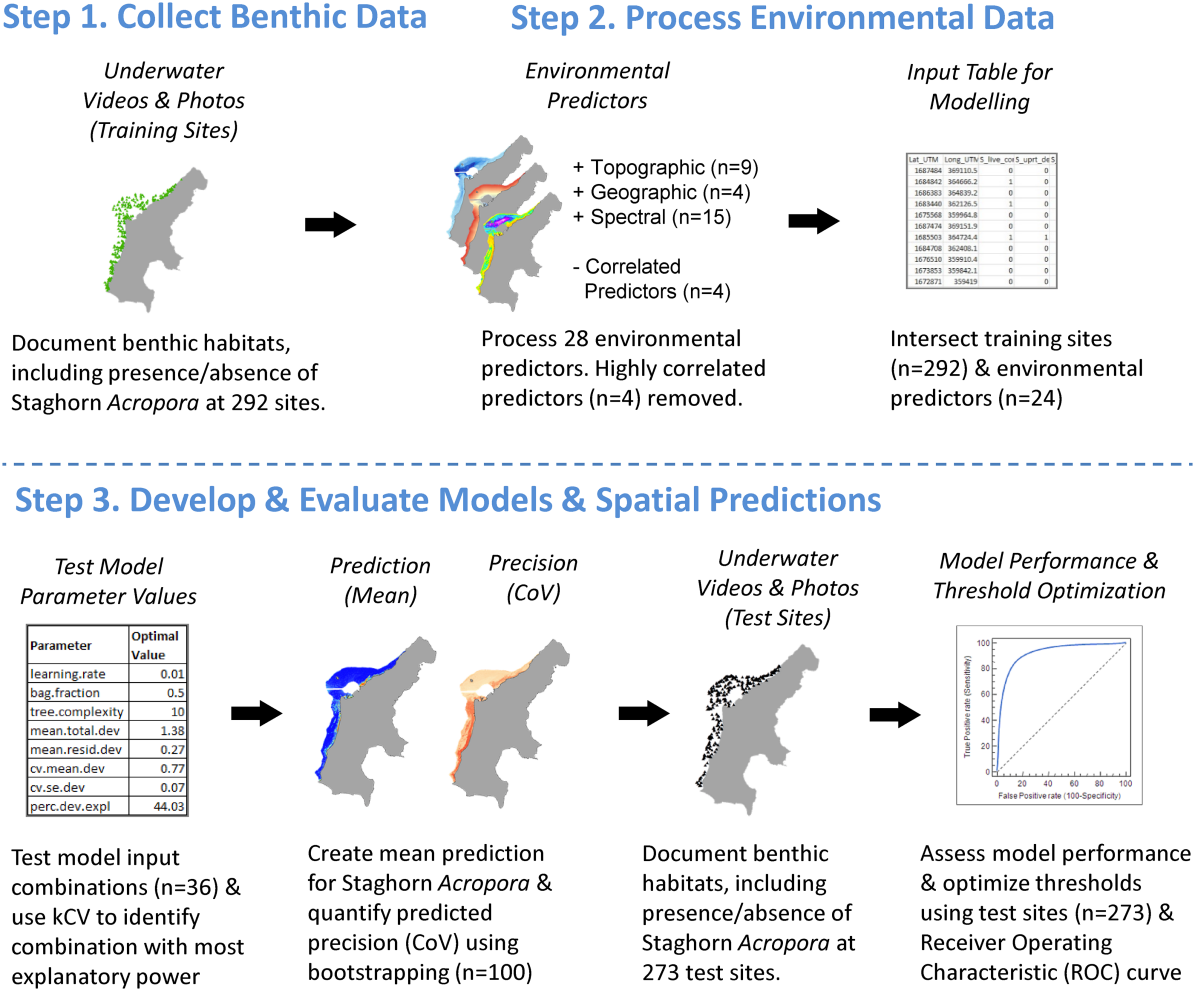
Predicting staghorn *Acropora*. Diagram depicting steps in modeling process to predict staghorn *Acropora* distributions and quantify the uncertainty associated with these predictions.

Given the ecological importance and extensive death of staghorn *Acropora* species, we developed a spatial prediction denoting the probability of occurrence for this coral community to help inform the SLUMP and subsequent management actions [21, 43]. This prediction was created using Boosted Regression Trees (BRTs). BRTs are mathematical techniques that can model complex, non-linear relationships between marine organisms and environmental variables [46, 47, 48]. We used this modeling technique because it is flexible, robust, and compares favorably to other modeling techniques [47, 48, 49, 50]. There were three main steps in the BRT modeling process (Fig 2), which were conducted primarily in ArcGIS 10.4 [51] and R software [52] using the dismo [53], and raster [54] packages. The first step was to collect underwater videos documenting the spatial distribution of benthic habitats, including the presence of live, upright staghorn *Acropora* throughout the lagoon [55]. Two hundred and ninety two sites were collected solely for training and optimizing the BRT models. An additional set of 273 sites were collected separately using a random stratified sampling design. These 273 sites were spatially independent, and used exclusively to test the performance of the model and accuracy of the spatial predictions.

The second step in the modelling process was to acquire remotely sensed data describing environmental conditions that could predict the distribution of staghorn *Acropora* in the lagoon. Twenty-eight environmental datasets (i.e., predictors) were acquired or derived on a 2x2 meter grid (S1–S4 Figs). Fifteen of these were spectral, and based on a WorldView 2 (WV2) satellite image acquired on February 5, 2016. Spectral information was included in the modeling process because it has been extensively and successfully used to map and characterize benthic habitats in tropical marine ecosystems, including the western Pacific [56, 57, 58]. Nine predictors described the topography of the seafloor derived from a depth surface. Seafloor depth and topography were included in the modeling process because they are useful predictors of geomorphological structure and benthic habitat types, such as sand, coral rubble, pavement, and coral reefs [59,60,61]. Four predictors described the geography (e.g., latitude, longitude, distance to key features) of the Lagoon, and were included as surrogates for other spatial information (e.g., oceanographic, ecological or anthropogenic) that were not readily available at 2x2 m. Geographic proxies were included in the modeling process because they are closely linked to environmental factors (e.g., temperature, turbidity) that directly influence species distributions [62], and have been successfully used to characterize the distribution of benthic habitats in past research efforts [60, 61, 63]. Values for these 28 predictors were spatially intersected with the locations of the underwater videos to create an input table for the BRT modeling process. Highly correlated predictors (i.e., spectral bands 2, 4, 6, 12) were dropped (Spearman Rank r ≥ 0.9 or r ≤ -0.9), and the remaining 24 environmental predictors were used in the modelling process.

The third step in the modeling process was to use this table to tune the BRT models, make predictions across space and quantify uncertainty associated with these predictions. Thirty-six model input combinations were tested during the tuning process, including three values for learning rate (*lr*), six values for tree complexity (*tc*), and two values for bag fraction (*bf*) (Table 1). These values were chosen based on expert opinion and previous research developing BRTs [48, 53], and tested using *gbm*.*step* function in the R dismo package [53]. K-fold (k = 10) cross validation (kCV) [48, 64] was used identify the values of *lr*, *tc*, and *bf*, which produced the “best” model. Here, the “best” model was defined as having the most explanatory power, quantified using percent deviance explained (PDE). PDE is the amount (%) of variation explained in the response data with the average trend removed. PDE values normally range between 0 and 100%, and models with higher PDEs were considered to have more explanatory power (and thus perform better) than models with lower PDEs. To identify this “best” model, 90% of the data (selected by bag fraction) were used to create the model, and the remaining 10% of the data were used to quantify PDE. This process was repeated 10 times to create 10 separate models with 10 separate PDEs for a single combination of *lr*, *tc* and *bf*. These 10 PDEs were averaged, and the combination of *lr*, *tc*, and *bf* that produced the highest average PDE was selected. After this selection was made, all models developed during this tuning step were discarded.

**Table 1:** Model parameters and values tested during the BRT tuning process. The combination of parameter values (i.e., *lc* = 0.005, *tc* = 3 and *bf* = 0.75) with the highest percent deviance explained were identified using 10-fold cross validation, and then used to create the spatial prediction for staghorn *Acropora* in Saipan Lagoon.

Model Parameter	Values Tested	Description	Impact
learning rate (*lr*)	0.01, 0.001, 0.005	Determines contribution of each tree to the growing model	Decreasing (slowing) lr increases the number of trees required for optimal prediction
tree complexity (*tc*)	2, 3, 4, 5, 10, 20	Controls how many predictor interactions are fitted in a tree	Decreasing tc will shrink the size (number of nodes) in a tree
bag fraction (*bf*)	0.5, 0.75	Controls proportion of data randomly selected to build each tree	Decreasing bf will reduce the number of points randomly used to build a tree

The above model tuning process identified the following optimal input values: *lr* = 0.005, *tc* = 3, *bf* = 0.75. New BRT models were then created using these optimal values and a random, data resampling technique called bootstrapping. Bootstrapping was used to create 100 separate models by randomly resampling (with replacement) the training sites used to fit the models. This iterative process was used to understand and quantify model variability and bias, and was implemented in R software using the *lapply* function. These 100 models were then used to create 100 separate probability of occurrence predictions (using the *predict* function in the R raster package [54]) for staghorn *Acropora* on a 2x2 meter grid. Probability of occurrence denotes the likelihood of finding staghorn *Acropora* in a particular 2x2 m cell. Larger probabilities indicate that staghorn *Acropora* is more likely to be present and vice versa. A mean staghorn *Acropora* prediction was calculated by averaging these 100 separate predictions in each 2x2 m cell. Precision was also calculated using these 100 separate predictions, and is reported as coefficient of variation (CoV). CoV is one of the primary measures of uncertainty described here. The performance and accuracy of this mean prediction was also evaluated using a separate set of spatially-independent sites (n = 273), which is discussed in more detail in the next section.

### Quantifying uncertainty in staghorn *Acropora* models

Many management needs require converting a grid-based map with continuous prediction values into a CHM showing predicted presence and absence. While CHMs are fixed and unchangeable, SDMs can be tailored to specific management needs or optimized to meet specific conservation objectives by changing their probability thresholds [1, 11]. For example, if managers want to understand the cost (in conservation terms) of over and under predicting a species distribution, then there are statistical tools and techniques (e.g., receiver operating characteristic curves) to identify the appropriate breakpoint in the SDMs outputs. If the amount of acceptable cost goes up or down, the thresholds can be changed and tuned accordingly. The ability to tune continuous maps and SDM outputs makes them more flexible than CHMs for many marine management applications [1, 11, 65]. However, it can be challenging to identify optimal probability thresholds, and even more so when the uncertainty (i.e., precision and performance) of the spatial prediction varies across geographic space.

Here, precision describes how much the staghorn *Acropora* prediction varies within a grid cell when given different, random input datasets. This variation was quantified using bootstrapping (n = 100) described above, and was reported as coefficient of variation (CoV). CoV is the unitless ratio of the standard deviation to the mean. It is a transparent way to quantify uncertainty, and can be translated into ranges of predicted probabilities. Instead of reporting two values (i.e., minimum and maximum), CoV captures this range of probabilities in a single value (i.e., 0.1). A CoV equal to 0.1 means that a probability could vary by 10%. If the probability for a grid cell is 50%, then 50% x 10% = ± 5%. If the probability is 80%, then 80% x 10% = ± 8% and so on. Smaller CoVs indicate that the prediction has higher precision and less uncertainty (and vice versa).

While precision describes variation in the prediction, it does not describe the accuracy or performance of the prediction. Here, performance describes the number and magnitude of errors in a species or assemblage prediction relative to its observed distribution *in situ*. The performance of the staghorn *Acropora* prediction was evaluated using the full independent test (n = 273) dataset to calculate Percent Deviance Explained (PDE) and Receiver Operating Characteristic (ROC) Area under the Curve (AUC) [21]. The PDE for the final staghorn Acropora model was 30% and the AUC was 0.93, both indicating good to excellent performance. PDE is the amount (%) of variation explained in the response data with the average trend removed. PDE values normally range between 0 and 100%, with higher values indicating better model performance and lower error. ROC curves measure a model’s predictive performance by comparing a model’s sensitivity to its specificity. Sensitivity and specificity quantify a model’s ability to correctly predict the presence and absence of a species, respectively [65]. These rates change based on the probability threshold that is chosen, denoting where staghorn *Acropora* is classified as present and absent.

Table 2 shows examples of how these rates change based on different probability thresholds. For the 29% probability threshold, 42% of the presences and 100% of the absences are correctly classified. However, this ratio shifts to 50% and 98% (respectively) when the probability threshold is decreased to 17%. It changes to 92% and 85% (respectively) when the probability threshold is decreased further to 4%. This shift (from correctly predicted absences to correctly predicted presences) occurs because, as the probability decreases, staghorn is predicted to occur in a larger geographic area, increasing the chances that it is correctly predicted as present. This tension between correctly predicted presences and absences makes consciously choosing the optimal probability threshold critical for each management application. Choosing one threshold over another can have significant impacts on the resulting products and any subsequent decisions made using those products. This impact is particularly pronounced for rare species [65], like staghorn *Acropora*.

**Table 2:** Performance of methods used to optimize the probability of occurrence threshold. Performance metrics included accuracy, sensitivity, specificity and Kappa. Note that the SES and MSS approaches resulted in the same threshold value (0.04).

Optimization Method	Probability of Occurrence Threshold	Accuracy (% Correctly Classified)	Sensitivity	Specificity	Kappa
SES	0.04	0.85	0.92	0.85	0.30
MSS	0.04	0.85	0.92	0.85	0.30
PPOP	0.17	0.96	0.50	0.98	0.48
Kappa	0.29	0.98	0.42	1.00	0.58

### Incorporating staghorn *Acropora* model uncertainty in marine zoning decisions

**Fig 3:**
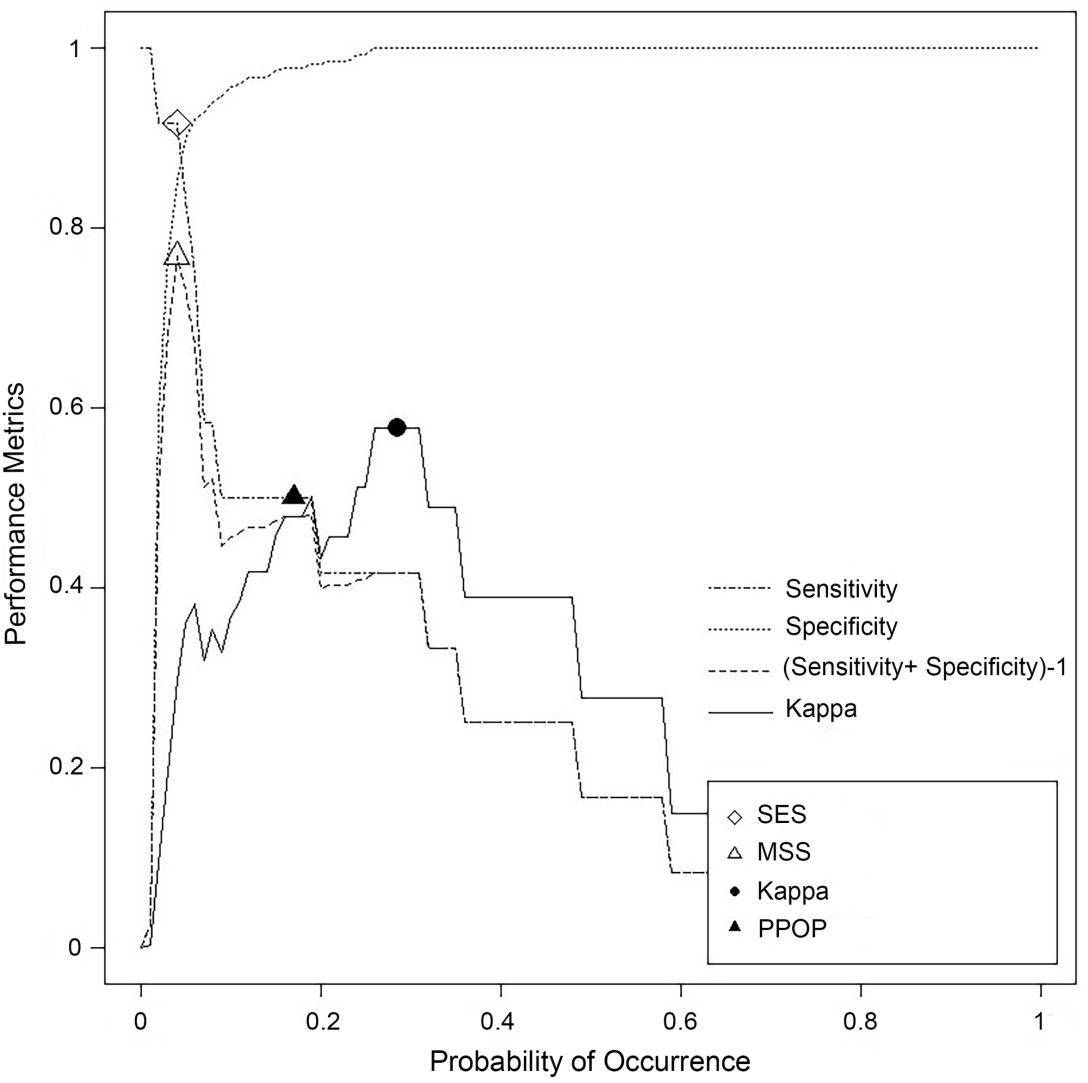
Optimizing probability of occurrence thresholds. Four methods were evaluated for choosing a threshold using a ROC curve, including SES, MSS, Kappa and PPOP. Plot created using ‘PresenceAbsence’ package in R software.

Different pairs of probability and precision thresholds were examined to help managers understand how changing these thresholds affected the staghorn *Acropora* prediction. For probability, there are several approaches for optimizing how a threshold is selected [65, 66, 67]. We examined four of the more commonly used approaches using ROC curves. The first approach, Sensitivity Equals Specificity (SES), identifies the threshold where presences and absences have an equal chance of being correctly predicted. The second approach, Maximum Sensitivity and Specificity (MSS), locates the threshold where presences and absences both have their maximum chance of being correctly predicted. The third approach, Predicted Prevalence equals Observed Prevalence (PPOP), pinpoints the threshold where the *in situ* prevalence of a species (e.g., 4.5% for staghorn *Acropora*) is equal to the predicted prevalence of a species. The fourth and final approach, Maximum kappa coefficient (Kappa), describes the threshold with the highest proportion of correctly classified test sites (after accounting for the likelihood of chance agreement). Using these techniques, we identified four “optimum” probability of occurrence thresholds (i.e., 0.04, 0.17, and 0.29) (Table 2; Fig 3). Only three thresholds were identified because the SES and MSS approaches identified the same breakpoint. This happens in some cases when model performance is good to excellent (i.e., when the ROC area under the curve ≥ 0.8). We then applied these three thresholds to reclassify the staghorn *Acropora* prediction into three separate presence/absence maps.

Next, we divided the CoV surfaces into terciles, so we could better understand the impacts of precision over a broad range of values. Thresholds for these terciles were based on the range of precisions found within the spatial footprints of each reclassified probability surface. Precisions ranged from CoV ≥ 0.10 to ≤ 0.85 ($\bar{\text{x}}=0.47\pm 0.12$) within the spatial footprint of the high probability surface. They ranged from CoV ≥ 0.10 ≤ 1.11 ($\bar{\text{x}}=0.61\pm 0.15$) for the moderate probability surface, and from CoV ≥ 0.10 ≤ 1.75 ($\bar{\text{x}}=0.91\pm 0.20$) for the low probability surface. These precisions describe the impact of having uncertainty up to 85%, 110% or 175% of the probability value in a grid cell. We reclassified the CoV surfaces into terciles corresponding to high, moderate, and low precisions. The three reclassified probability maps and reclassified precision maps were then merged to create nine maps showing how uncertainty changes the predicted presence/absence of staghorn *Acropora* in the lagoon (Table 3).

**Table 3:** Combinations of probability and precision thresholds. Nine staghorn *Acropora* presence/absence maps were created using these nine combinations of probability and precision.

		Probability of Occurrence		
		Low	Moderate	High
Precision	Low	p≥0.04, CoV≤1.75	p≥0.17, CoV≤1.11	p≥0.29, CoV≤0.85
	Moderate	p≥0.04, CoV≤0.99	p≥0.17, CoV≤0.67	p≥0.29, CoV≤0.54
	High	p≥0.04, CoV≤0.83	p≥0.17, CoV≤0.56	p≥0.29, CoV≤0.43

Lastly, we quantified how the location and size of geographic areas predicted to contain staghorn *Acropora* changed in these nine combinations. These spatial metrics were chosen because they were explicit and easily calculated in the context of the existing jet ski exclusion zones and potential jet ski operation zones and transit corridors. They are also ecologically relevant because the location and size of protected areas can influence the ecological function and resilience of marine communities [68]. Smaller, more fragmented and spatially distributed habitats may not serve the same ecological function or be as resilient as larger, more connected marine areas. Combined, these metrics and maps translate complex SDMs into products that more simply illustrate how staghorn *Acropora* predictions change based on uncertainty. They also provide baseline maps and the basis for explicit management targets that can be used to evaluate boundary alternatives for the existing jet ski exclusion zones and potential jet ski operation zones.

## Results

### Impact of Uncertainty on staghorn acropora predictions in the Lagoon

**Fig 4:**
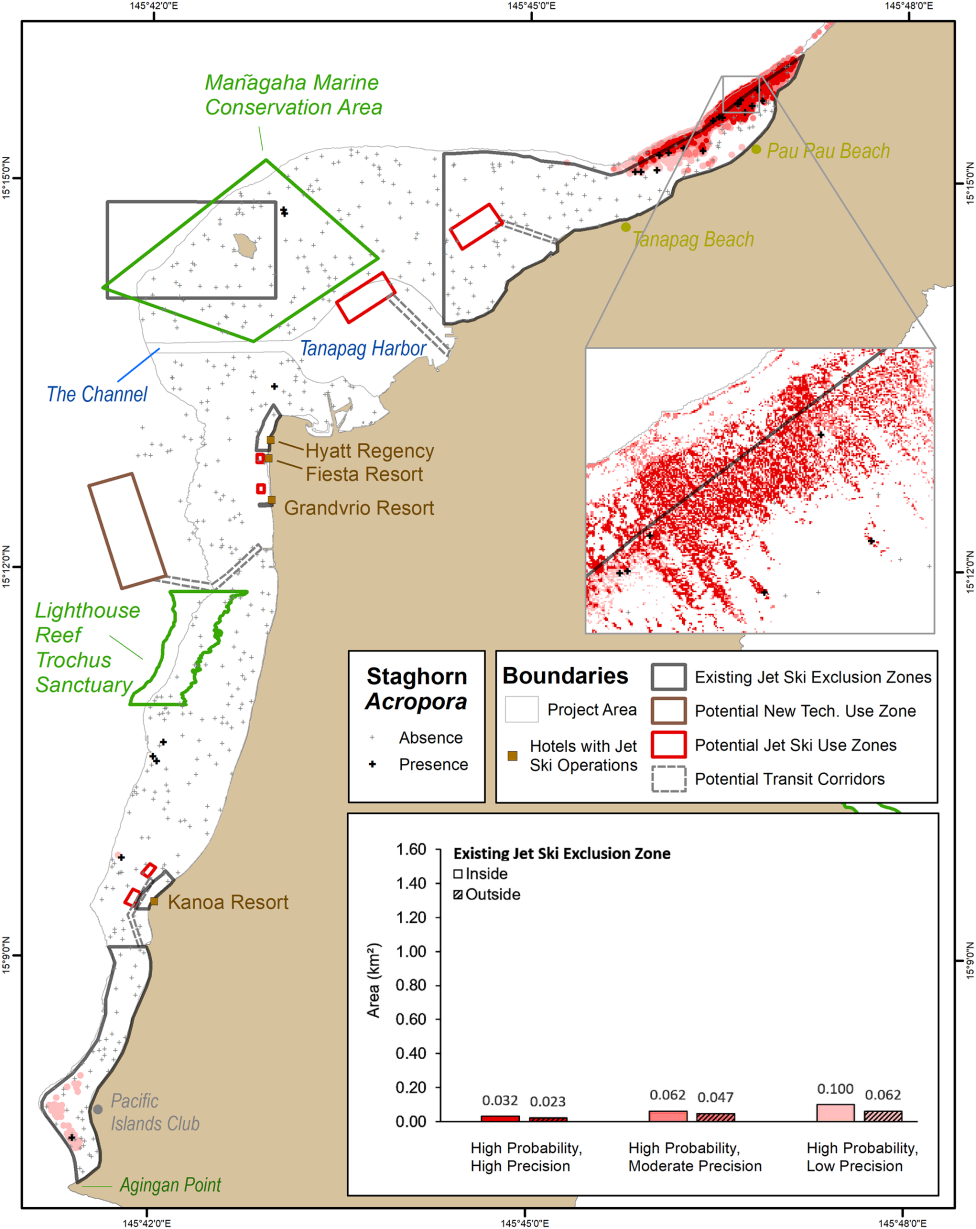
Locations where staghorn *Acropora* have a higher probability (0.29) of occurring. These high probability locations are divided based on high, moderate and low precisions. The bar graph depicts the amount of area (km^2^) inside and outside the existing jet ski exclusion zones predicted to have staghorn *Acropora*. The number above each bar denote the predicted area in km^2^. The colors and groups in the bar graph match the colors and groups in the map.

**Fig 5:**
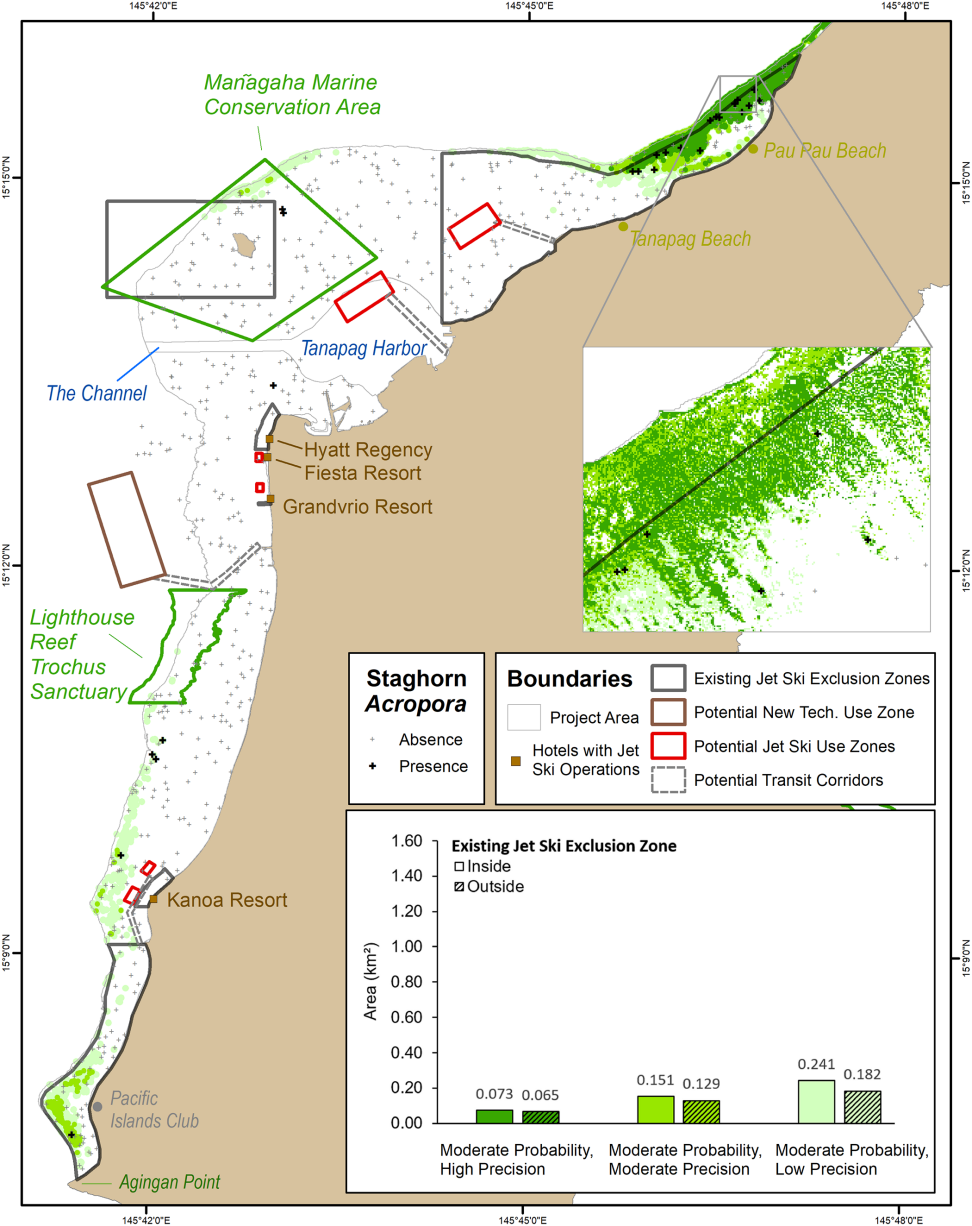
Locations where staghorn *Acropora* have a moderate probability (0.17) of occurring. These moderate probability locations are divided based on high, moderate and low precisions. The bar graph depicts the amount of area (km^2^) inside and outside the existing jet ski exclusion zone predicted to have staghorn *Acropora*. The number above each bar denote the predicted area in km^2^. The colors and groups in the bar graph match the colors and groups in the map.

**Fig 6:**
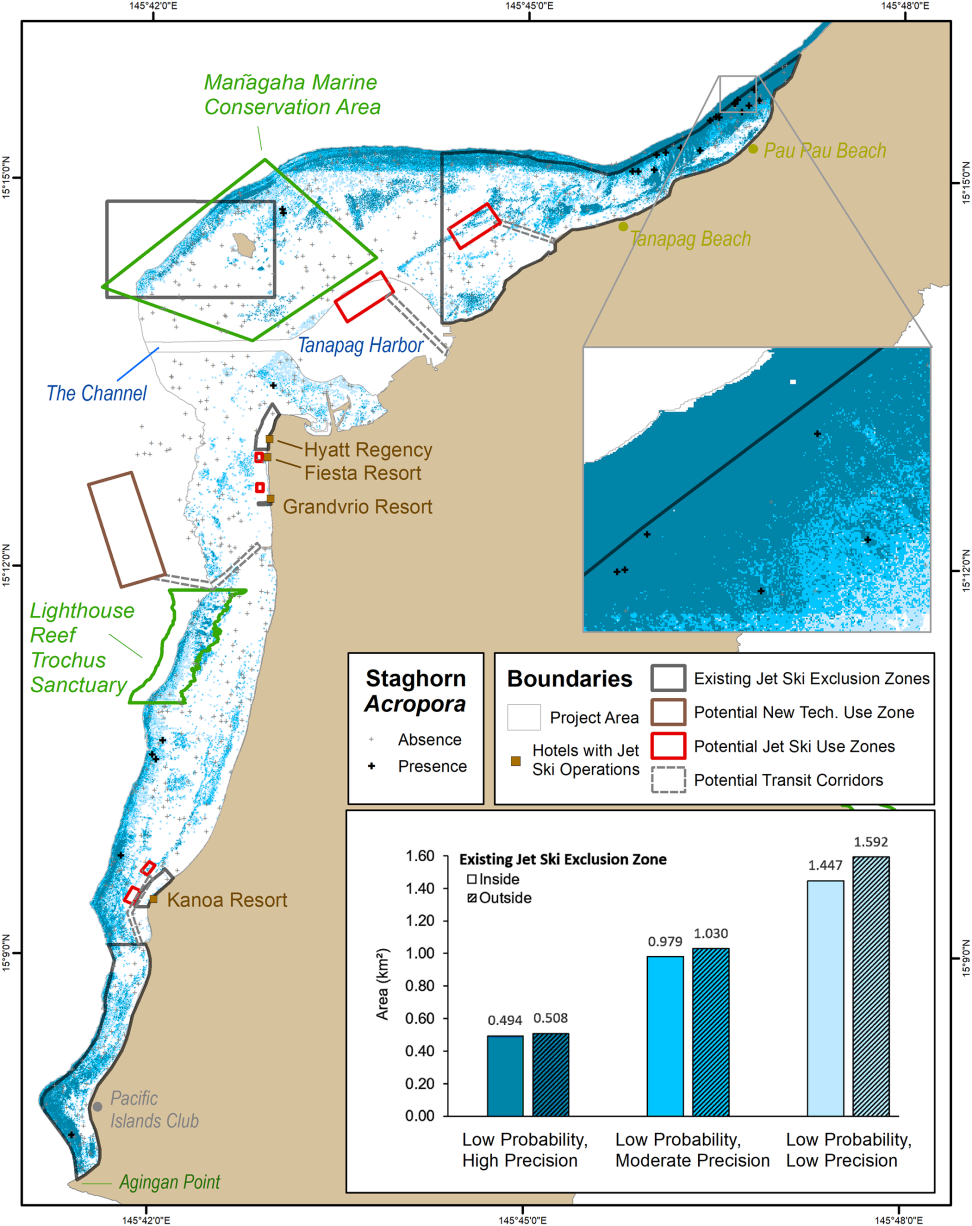
Locations where staghorn *Acropora* have a low probability (0.04) of occurring. These low probability locations are divided based on high, moderate and low precisions. The bar graph depicts the amount of area (km^2^) inside and outside the existing jet ski exclusion zone predicted to have staghorn *Acropora*. The number above each bar denote the predicted area in km^2^. The colors and groups in the bar graph match the colors and groups in the map.

Inside Saipan Lagoon, the nine uncertainty combinations (Table 3) substantially affected the size (km^2^) of the geographic areas predicted to contain staghorn *Acropora* (Figs 4,5 and 6). Overall, the total amount of predicted area (in the entire Lagoon) increased as probability of occurrence and precision thresholds decreased. The magnitude of these changes varied across the nine uncertainty combinations, and were calculated by dividing the amount of predicted area (km^2^) for each uncertainty combination. Specifically, the moderate probability and precision combination predicted approximately 5x more geographic area with staghorn *Acropora* than the highest probability and precision combination (i.e., 0.28 km^2^/0.05 km^2^ ~ 5). The low probability and precision combination predicted approximately 11x more geographic area with staghorn *Acropora* than the moderate probability and precision combination (i.e., 3.04 km^2^/0.28 km^2^ ~ 11), and 56x more geographic area than the highest probability and precision combination (i.e., 3.04 km^2^/0.05 km^2^ ~ 56). Between these uncertainty combinations, it is unknown whether there are similar differences in the amount of predicted area for staghorn *Acropora*.

The nine uncertainty combinations also had a substantial effect on the location of the geographic areas predicted to contain staghorn *Acropora* (Figs 4, 5 and 6). Specifically, staghorn presence expanded southward along the reef crest as the uncertainty thresholds decreased (Figs 4, 5 and 6). For the highest probability and precision thresholds, staghorn *Acropora* predicted presence was concentrated north of Tanapag Beach. These maps encapsulated the core geographic area and the highest concentration of observed staghorn *Acropora* presences. However, these maps also excluded approximately six sites where staghorn *Acropora* was present elsewhere in the lagoon, particularly south of Lighthouse Reef Trochus Sanctuary. The predicted geographic extent of staghorn was much broader for the lowest probability and precision thresholds. Predicted staghorn extended offshore of Pau Pau Beach along the reef crest to the Mañagaha Marine Conservation Area, and along the reef crest from the Lighthouse Reef Trochus Sanctuary south to Agingan Point. These lowest probability and precision maps encapsulated all 25 sites with observed staghorn presences. However, they also incorrectly included many more sites where staghorn *Acropora* was found to be absent. These patterns indicate that calculating the location and amount of potential live staghorn *Acropora* is sensitive to the probability and precision thresholds that are applied.

### Impact of prediction uncertainty inside the existing jet ski exclusion zones

There are six existing jet ski exclusion zones encompassing approximately 12 km^2^ of area in the lagoon. Of all the sites surveyed in the lagoon, 68% (n = 17/25) of the sites with staghorn *Acropora*, and 40% (n = 112/279) of the sites without staghorn *Acropora* were located inside these zones. For all nine uncertainty combinations, the amount and location of area predicted to contain staghorn *Acropora* varied inside these zones (Figs 4, 5, 6). Overall, the high probability thresholds predicted the least amount of live staghorn coral in the fewest number of exclusion zones (Fig 4). Specifically, the high probability and high precision threshold predicted the presence of live staghorn coral in only one zone, offshore of Pau Pau Beach. Live staghorn coral was predicted to be absent in the remaining five zones. Spatial patterns were different for the map developed using the high probability and moderate precision threshold. This uncertainty combination predicted the presence of staghorn over twice as much geographic area than the previous threshold, although it was located in the same northernmost zone. The last uncertainty combination (i.e., high probability and low precision) predicted approximately 62% more live staghorn coral than the moderate precision threshold over a broader geographic area. These additional predicted areas were located in the northernmost zone and the southernmost zone between the Pacific Islands Club and Agingan Point. Live staghorn coral was predicted to be absent in the remaining four zones.

Next, the moderate probability thresholds predicted a larger amount of live staghorn coral present in a greater number of exclusion zones than the high probability thresholds (Fig 5). The moderate probability and high precision threshold predicted staghorn presence in two zones, comprising 0.07 km^2^ of area. These predictions were concentrated in the northernmost zone, offshore of Pau Pau Beach and in the southernmost zone between Pacific Islands Club and Agingan Point. Live staghorn coral was predicted to be absent in the remaining four zones. Spatial patterns were different in the map developed using the moderate probability and moderate precision threshold. This threshold combination predicted the presence of staghorn over twice as much geographic area and in an additional exclusion zone than the previous threshold. The additional predicted live staghorn coral was located in the northernmost zone, southernmost zone and the zone around Mañagaha Island. Live staghorn coral was predicted to be absent in the remaining three zones offshore of the Hyatt Regency, Grandvrio Resort and Kanoa Resort. The last uncertainty combination (i.e., moderate probability and low precision) predicted approximately 60% more live staghorn coral than the previous threshold, although this additional area was concentrated in the same three exclusion zones as the moderate precision threshold.

Lastly, the low probability threshold combinations predicted the largest amount of live staghorn coral in the greatest number of exclusion zones (Fig 6). Like above, the amount of area predicted to contain staghorn Acropora increased as the precision decreased. Specifically, the moderate precision threshold predicted twice as much geographic area as the high precision threshold, and the low precision threshold predicted 49% more live staghorn coral than the moderate precision threshold. Despite these differences, the predicted location of staghorn *Acropora* occurred in the same four exclusion zones for all three low probability thresholds. These predictions were concentrated in the northernmost zone, offshore of Pau Pau Beach, along the reef crest in the zone around Mañagaha Island, in the zone offshore of the Kanoa Resort and in the southernmost zone between Pacific Islands Club and Agingan Point. Live staghorn coral was predicted to be absent in the remaining two zones offshore of the Hyatt Regency and Grandvrio Resort. These patterns suggest that, inside the existing exclusion zones, the amount and location of staghorn *Acropora* is sensitive to the uncertainty thresholds that are applied. These relationships are inversely proportional, since the amount of predicted staghorn *Acropora* and the number of zones (where staghorn was predicted to occur) increased as the uncertainty thresholds decreased.

### Impact of prediction uncertainty outside the existing jet ski exclusion zone

In addition to affecting the results inside the exclusion zones, the uncertainty thresholds also affected the amount and location of predicted live staghorn coral outside the existing, six exclusion zones. Overall, the high probability thresholds predicted the least amount of live staghorn coral in geographically concentrated areas outside the existing zones (Fig 4). The high probability and high precision threshold predicted staghorn presence outside the northernmost zone, comprising 0.02 km^2^ of area. These predicted areas were located landward of the reef crest, which are periodically exposed at low tide and inaccessible by jet skis. Spatial patterns were different in the map developed using the high probability and moderate precision threshold. This uncertainty combination predicted staghorn presence over twice as much geographic area seaward of the northernmost zone. The last uncertainty combination (i.e., high probability and low precision) predicted approximately 32% more live staghorn coral outside the existing zones than the moderate precision threshold. These additional predicted areas were also located seaward of the northernmost zone, however they also extended further south along the reef crest towards Tanapag Beach. Live staghorn coral was predicted to be absent elsewhere outside the existing zones in the lagoon for these three uncertainty combinations.

The moderate probability thresholds predicted more staghorn *Acropora* outside the existing zones than the high probability thresholds. Unlike for the high probability thresholds, these additional predicted areas were spread out through the entire lagoon (Fig 5). The moderate probability and high precision threshold predicted staghorn presence outside the northernmost zone, comprising 0.07km^2^ of area. These predicted areas were clustered offshore of Pau Pau Beach and in isolated areas offshore of the Pacific Island Club. Spatial patterns were different in the map developed using the moderate probability and moderate precision threshold. This uncertainty combination predicted the presence of staghorn over twice as much geographic area than the high precision threshold. These predicted areas were located in few additional locations, including due north of Mañagaha Island along the reef crest, and offshore of the Kanoa Resort. The last uncertainty combination (i.e., moderate probability and low precision) predicted approximately 41% more live staghorn coral outside the existing zones than the moderate precision threshold. These predicted areas extended along the reef crest just outside the northernmost boundary, along the reef crest north of Mañagaha Island and from Lighthouse Reef Trochus Sanctuary south to the Kanoa Resort. Live staghorn coral was predicted to be absent elsewhere outside the existing zones for these three uncertainty thresholds.

Lastly, the low probability thresholds predicted the largest amount of staghorn *Acropora* outside the existing zones (Fig 6). These additional predicted areas were also much more geographically spread out compared to the other thresholds. The low probability and high precision threshold predicted the presence of live staghorn coral outside the northernmost zone, comprising 0.51 km^2^ of area. These predicted areas were not located near the reef crest or back reefs, like for the moderate probability thresholds. Rather, this threshold predicted live staghorn coral surrounding Mañagaha Island, south of Tanapag Harbor, offshore of the Hyatt Regency and Grandvrio Resort south to the Kanoa Resort. Spatial patterns were different in the map developed using the low probability and moderate precision threshold. This threshold combination predicted the presence of staghorn over twice as much geographic area than the high precision threshold. These predicted areas included all the same locations as above, as well as additional areas closer to the channel. The last uncertainty combination (i.e., low probability and low precision) predicted approximately 54% more live staghorn coral outside the existing zones than the moderate precision threshold. These predicted areas included all the same locations as above, as well as deeper areas closer to the channel and Tanapag harbor. Many of these areas are easily and readily accessible by jet skis. These patterns suggest that, outside the existing exclusion zones, the amount and location of potential staghorn *Acropora* are also sensitive to the uncertainty thresholds that are applied. These relationships are inversely proportional, since the amount and geographic extent of predicted staghorn *Acropora* increased as the probability and precision thresholds decreased.

### Impact of prediction uncertainty on potential jet ski operation zones

The updated SLUMP [44] identifies six potential jet ski operation zones encompassing 0.55 km^2^ of area in the lagoon. Transit corridors connect these jet ski operation zones with the nearest access point for jet ski users. While these potential zones and corridors contained some live coral (e.g., *Isopora palifera*), no staghorn *Acropora* was observed at the sites surveyed inside these potential zones or corridors. The six high and moderate probability maps showed the same trend, predicting that staghorn *Acropora* was absent inside all of the potential operation zones and corridors. However, the three low probability maps showed a different pattern. All three low probability combinations predicted live staghorn coral in two of the six potential zones (Table 4). These two potential zones were located west of Tanapag Beach and northwest of the Kanoa Resort. The low probability and low precision combination also predicted the presence of staghorn in the southernmost potential zone, offshore of the Kanoa Resort. These patterns suggest that calculating the amount and location of potential staghorn *Acropora* inside potential operational areas is also sensitive to the uncertainty thresholds that are applied. This relationship is inversely proportional, like with the existing exclusion zones. As the probability and precision thresholds decreased, staghorn *Acropora* was predicted in a greater number of potential areas.

**Table 4:** Predicted staghorn acropora inside proposed jet ski zones. Amount (m^2^) of predicted staghorn *Acropora* inside the proposed jet ski operation zones. Proposed jet ski operation zones are sequentially numbered from north (#1) to south (#6).

		Amount (m^2^) of Staghorn *Acropora*			
Proposed Jet Ski Operation Zones	Zone Size (km^2^)	Low Probability, High Precision	Low Probability, Moderate Precision	Low Probability, Low Precision	All other Uncertainty Combinations
1	219,010	1,159	2,588	3,349	0
2	262,069	0	0	0	0
3	10,035	0	0	0	0
4	10,200	0	0	0	0
5	17,854	21	134	193	0
6	28,293	0	0	13	0

### Staghorn acropora predictions and proximity to hotels with jet ski operations

**Fig 7:**
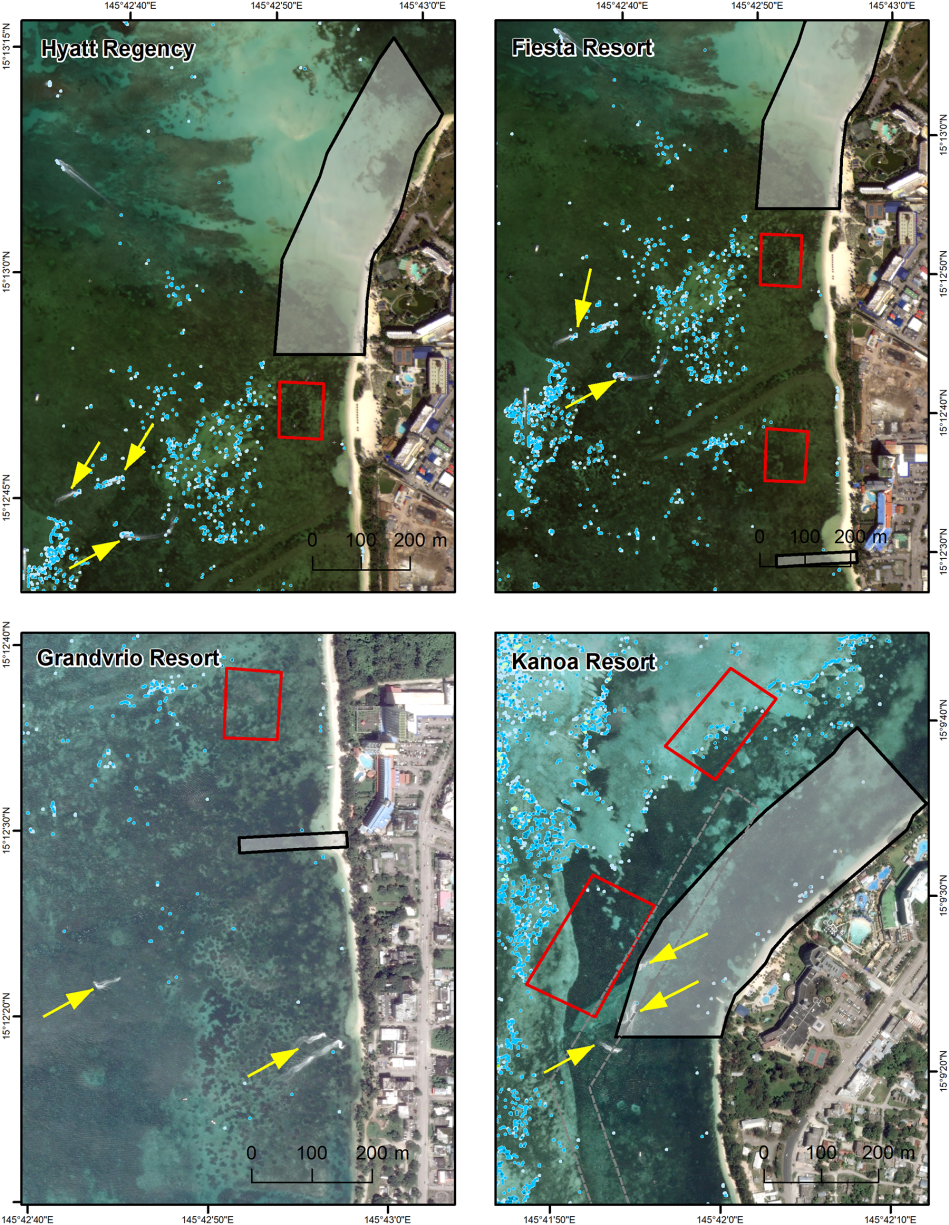
Jet ski activity offshore of four hotels in Saipan. The hotels include (clockwise) the Hyatt Regency, Fiesta Resort (satellite image taken 2/5/2016), Grandvrio Resort (11/7/2013), and Kanoa Resort (3/5/2013). The yellow arrows point out people using jet skis. The black boxes denote the existing jet ski exclusion areas, and the red boxes show the potential jet ski operation areas. The light to dark blue pixels denote locations where staghorn *Acropora* is predicted to be present (based on low probability and low, medium and high precisions, respectively).

In Saipan Lagoon, the Hyatt Regency, Grandvrio Resort, Fiesta Resort, and Kanoa Resort have commercial jet ski courses permitted in front of their beaches. Only one of the uncertainty combinations predicted that staghorn *Acropora* was near (<350 m) hotels with jet ski operations (Fig 7). Specifically, the low probability and low precision threshold predicted that staghorn *Acropora* occurred offshore the Hyatt Regency, Fiesta Resort, Grandvrio Resort, and Kanoa Resort. These predicted locations consist mainly of isolated grid cells, except for the area nearby the Kanoa Resort. This area had clusters of grid cells with low precisions and low to moderate probabilities approximately 600 meters offshore. Jet ski activity (identified by yellow arrows) can be seen offshore of these hotels in a few satellite images from 2013 and 2016 (Fig 7). In a few cases, jet skis are seen operating where staghorn *Acropora* is predicted to be present, offshore of the Hyatt Regency. In another case, two jet skiers can be seen operating inside the existing jet ski exclusion zone, offshore of the Kanoa Resort.

## Discussion

The framework described here was designed to illustrate how SDMs and associated uncertainty (for staghorn *Acropora*) can be translated into products for a targeted management application. It was also designed to help managers visualize the potential impacts of uncertainty on a specific marine zoning decision related to jet ski operations. However, this case study was not designed to make a single zoning recommendation, since that recommendation will depend on the level of uncertainty local managers are willing to accept in Saipan Lagoon. Our analysis showed that the amount of area and geographic location of predicted staghorn *Acropora* presence changed based on the nine uncertainty combinations. These changes in turn affected the amount of staghorn *Acropora* protected by the existing jet ski exclusion zones and the potential jet ski operation zones and transit corridors. Specifically, the lowest probability and lowest precision combination (i.e., those areas where the model predicted a >4% chance of *Acropora* being present ±175%) predicted that staghorn was present in five of the six existing exclusion zones, while the highest probability and highest precision combination predicted that it was present in only one zone. A manager could, consequently, consider opening up between two to five (of the six) existing exclusion zones to jet skis depending on the uncertainty threshold applied. Using this same analysis, a manager could also consider creating several new jet ski exclusion zones inside the lagoon. The location and size of these new areas would again depend on the uncertainty threshold applied. The low probability and low precision combination predicted live staghorn coral in several locations outside the existing exclusion zones, while the higher probability and higher precision combination predicted small amounts of live staghorn coral in one core location. For the higher thresholds, it is unlikely the predicted areas would need additional protection, since they were located in places too shallow for jet skis. However, for the lowest thresholds, the predicted areas were located in areas that are readily accessible by jet skis, including near hotels with commercial jet ski operations. These locations may be a higher priority for additional protections in Saipan Lagoon.

The effect of uncertainty thresholds was similar for the potential jet ski operation zones and transit corridors. The higher probability and higher precision combination (i.e., those areas where the model predicted a >29% chance of *Acropora* being present ±43%) predicted that staghorn *Acropora* was absent from all six of the potential operation zones or transit corridors. A manager using this threshold combination could reasonably conclude that jet ski operations in these areas would have minimal direct impacts on staghorn corals (since they are absent from these potential zones). Conversely, the low probability and associated precision thresholds predicted the presence of staghorn *Acropora* in three of the six potential operation zones and transit corridors. A manager using these uncertainty combinations would come to a very different conclusion, and may consider conducting targeted field surveys in these three potential zones to look for staghorn *Acropora* before permitting the use of jet skis. They may also consider relocating the three potential jet ski operation zones elsewhere, so they can be confident that they do not contain staghorn corals. In either situation, accepting one level of uncertainty over another may lead a manager to different conclusion and course of action when permitting jet ski exclusion or operation zones.

The above patterns and examples illustrate that uncertainty thresholds matter when using SDMs to make management decisions. These patterns and examples also suggest that each probability and precision combination has a tradeoff, and consequently, some combinations may be generally better suited for specific management applications. For example, staghorn *Acropora* predictions with high probability and higher precisions are better suited for management actions, monitoring efforts and outreach activities that are site-based and coral-specific. These thresholds are better suited to these types of applications because they will increase the likelihood of identifying core area with live staghorn *Acropora* for education opportunities and future study. Conversely, staghorn *Acropora* predictions with the lowest probability and lowest precisions may be better suited for actions where managers want to implement a precautionary principle, such as excluding jet skis use or protecting 30% of a habitat as in the Tulloch et al. 2013 example [4]. These thresholds are better suited to these types of applications because they will increase the likelihood that priority areas with live staghorn *Acropora* are not damaged by nearby ocean activities, and reduce the likelihood of injury to both the vessel operators and coral communities. Lastly, thresholds with higher probabilities and lower precisions (i.e., those combinations that predicted a >29% chance of *Acropora* being present ±83%) may be better suited for prioritizing future data collection efforts. These threshold combinations are better suited to these types of applications because the predictions in these locations would benefit most from the collection of additional *in situ* information about staghorn *Acropora* species. This additional information could be used to improve new models and future spatial predictions.

At the core of these different applications is the need to understand the staghorn *Acropora* prediction’s sensitivity to changing thresholds of uncertainty. As our results suggest, the size and location of priority areas may change when uncertainty is included in the decision making process. These spatial changes will impact the size and location of jet ski exclusion and operation zones for the protection of staghorn *Acropora*. More area should be protected if the lowest probability and lowest precision thresholds are used, while existing exclusion areas could be abolished if the higher probability and higher precision thresholds are used by managers in the lagoon. These findings are consistent with other research that has examined the impact of uncertainty on decisions in the marine environment [4, 69]. This growing body of research suggests that marine decision-making processes that exclude uncertainty may not achieve their marine management goals and conservation outcomes [4]. Identifying these management and conservation goals (ideally before data is collected) is critical for selecting the best data collection approach and defining acceptable levels of uncertainty associated with species distribution models.

## Conclusions

Species distribution models are often created to support non-technical managers and inform their decisions in the marine environment [69, 70, 71, 72, 73]. However, examples of these models being explicitly used to solve real-world marine management issues are sparse or the direct impact is intangible [1]. This disconnect means lost opportunities for the marine scientists to help answer real management questions [1, 17], and lost opportunities for managers to have the best available information [1, 18, 74]. One possibility for this disconnect is that SDMs are complex and nuanced, making them challenging for non-technical managers to use confidently [1, 18]. These challenges extend to understanding and applying the uncertainty associated with SDMs. Marine scientists need to spend more time and effort translating these outputs into simple products that can be immediately applied and more easily and universally understood. The work presented here uses staghorn *Acropora* and jet skis as an example to understand how uncertainty can be visualized in simple maps, and how it can impact management decisions. This work is one small step towards narrowing the gap between managers and scientists. In reality, these types of decisions are much more complex, and will be based on other considerations as well, including impacts to other sensitive habitats (e.g., seagrass), distance from hotels, safety of the jet ski operators and other ocean users, the cost of enforcement, and the level stakeholder support. Marine managers can help continue to grow and improve this process by working more closely and communicating more frequently with marine scientists about the finer details of their decision-making processes. Over the long term, closer partnerships and better communication may lead to better SDM outputs, more informed decisions, and more favorable marine conservation outcomes.

## Supporting information

S1 Fig Spectral predictors. Maps depicting the orthorectified, atmospherically, and water-column corrected WV2 band pairs (1–6) used to create map products.
(TIFF)

S2 Fig Spectral predictors (Continued). Maps depicting the orthorectified, atmospherically, and water-column corrected WV2 band pairs (7–15) used to create map products.
(TIFF)

S3 Fig Topographic predictors. Maps derived from the SD depth surface depicting the topography of the seafloor and used to create map products.
(TIFF)

S4 Fig Geographic predictors. Maps depicting the geographic predictors used to create map products.
(TIFF)
