# Supplementary figures and images for: Managers, modelers, and measuring the impact of species distribution model uncertainty on marine zoning decisions
Source: PLoS One. 2018 Oct 10;13(10):e0204569. doi: 10.1371/journal.pone.0204569 (PMC6179233; doi:10.1371/journal.pone.0204569)

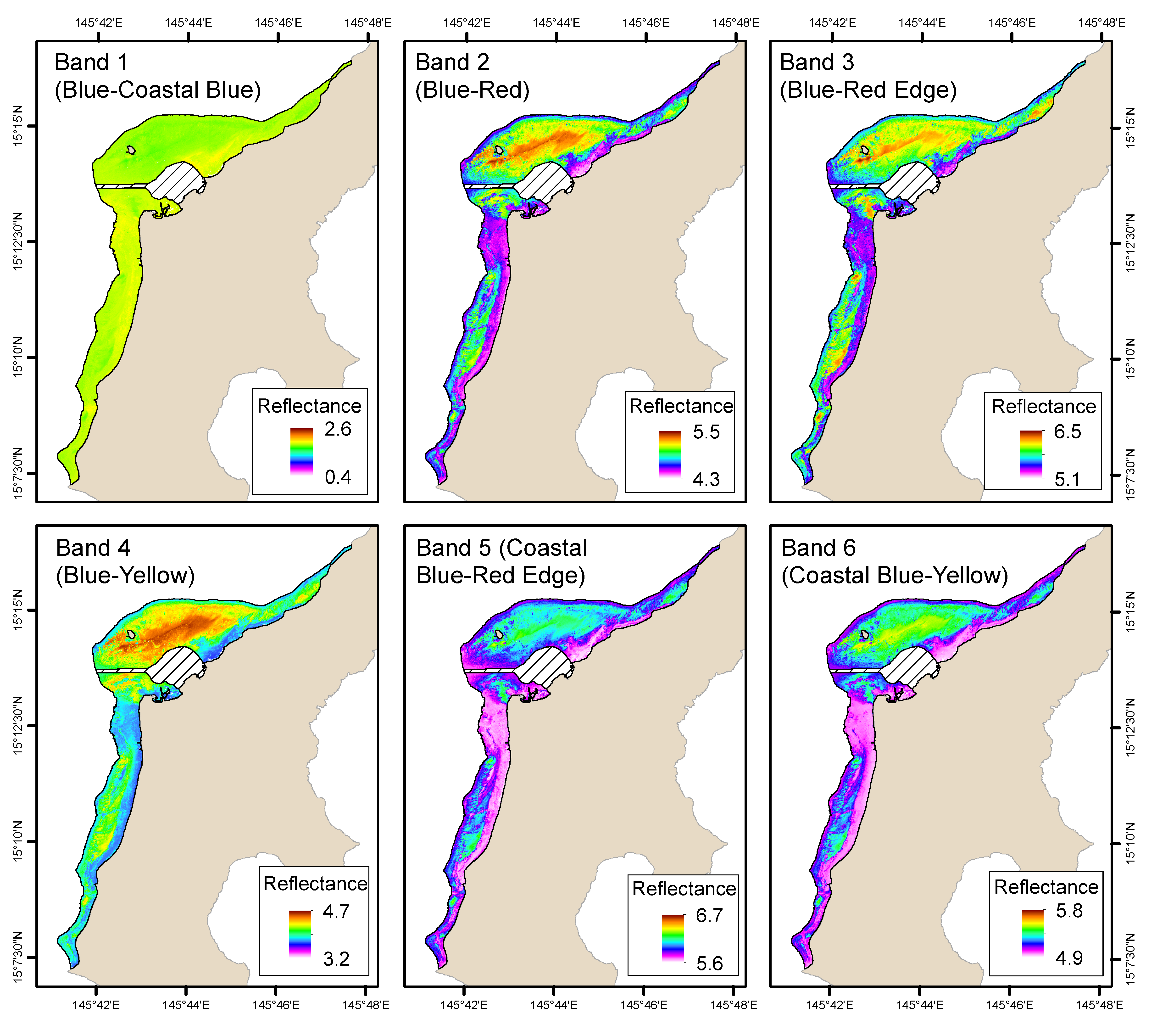

Supplement: S1 Fig — Maps depicting the orthorectified, atmospherically, and water-column corrected WV2 band pairs (1–6) used to create map products. (TIFF) [file pone.0204569.s001.tiff]

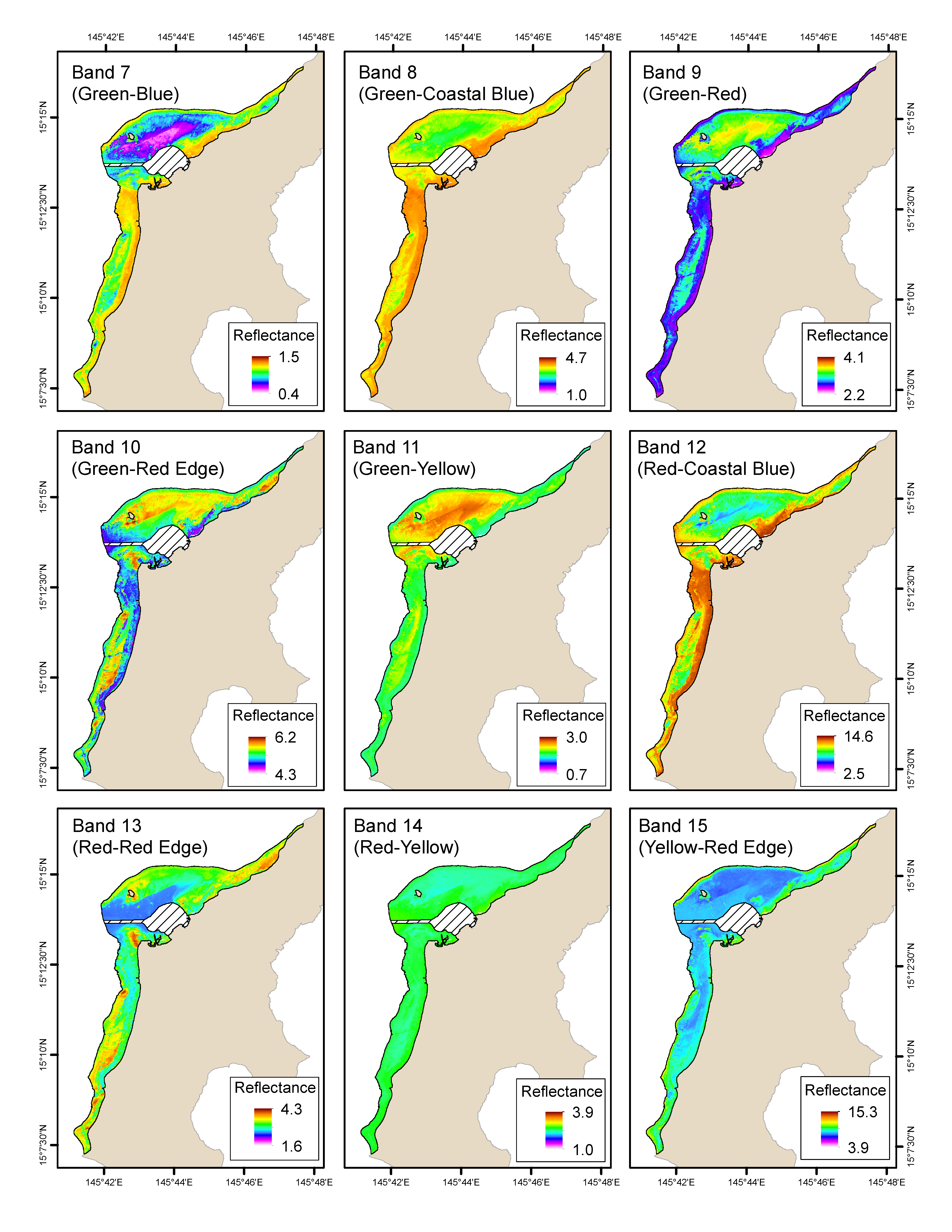

Supplement: S2 Fig — Maps depicting the orthorectified, atmospherically, and water-column corrected WV2 band pairs (7–15) used to create map products. (TIFF) [file pone.0204569.s002.tiff]

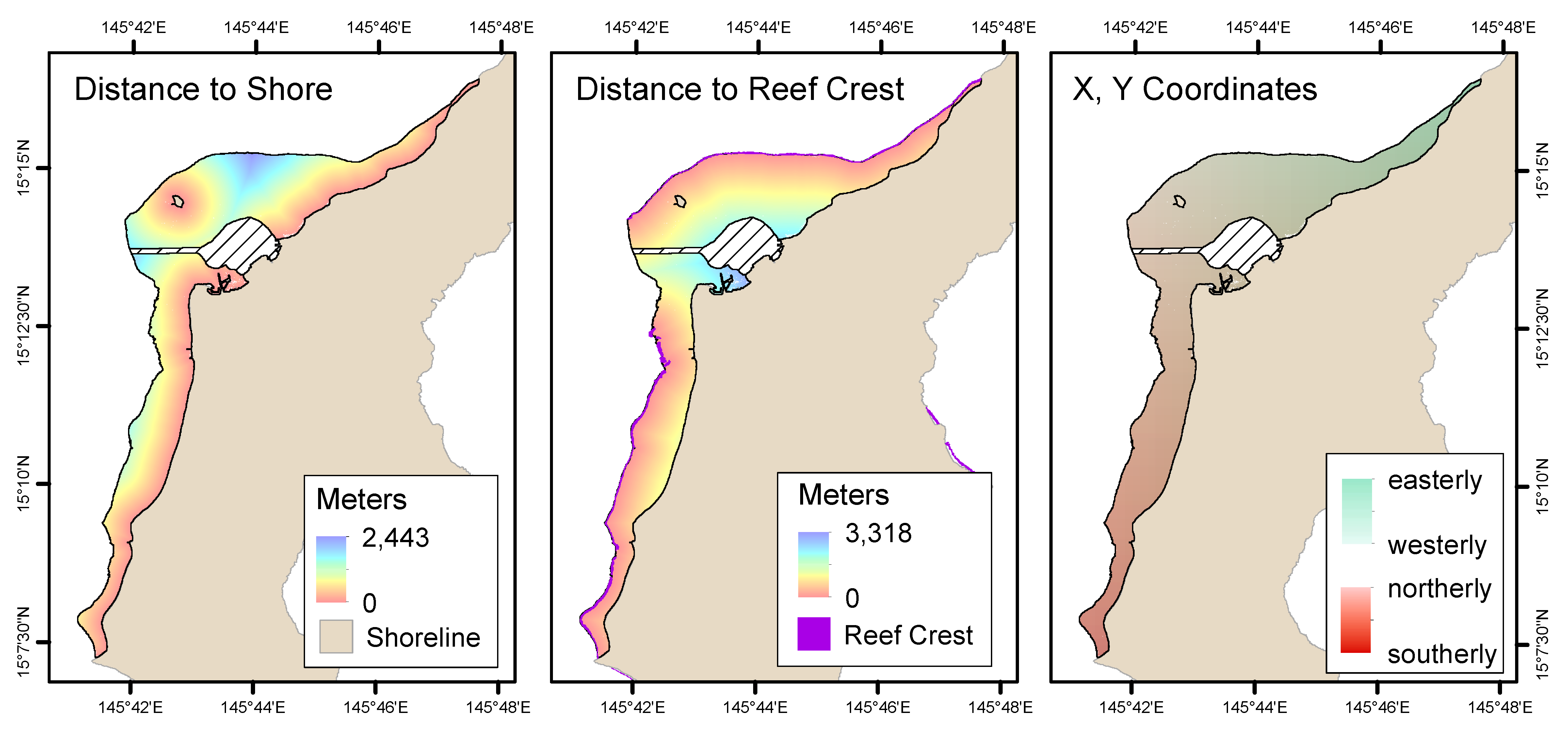

Supplement: S3 Fig — Maps derived from the SD depth surface depicting the topography of the seafloor and used to create map products. (TIFF) [file pone.0204569.s003.tiff]

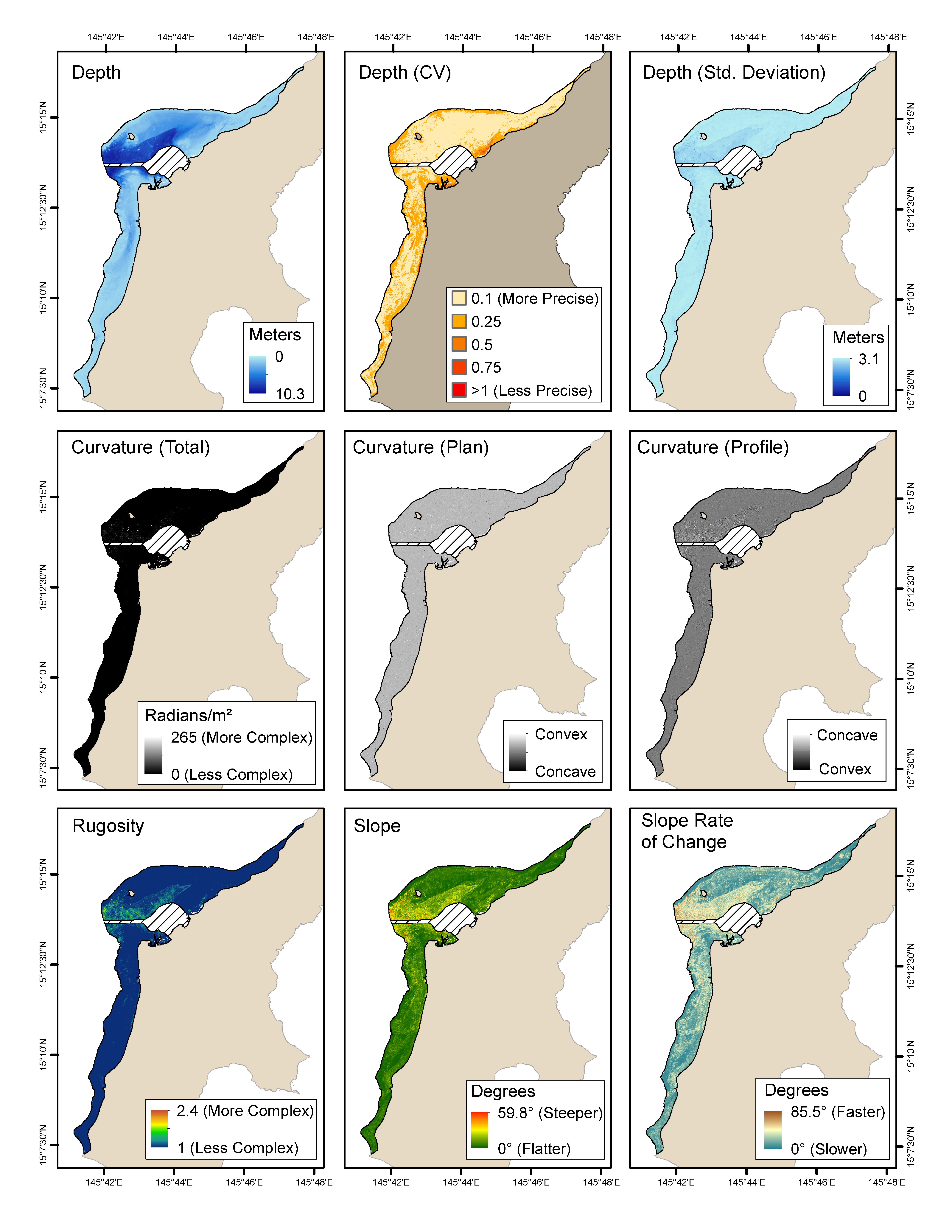

Supplement: S4 Fig — Maps depicting the geographic predictors used to create map products. (TIFF) [file pone.0204569.s004.tiff]
